# Dynamic distribution of gut microbiota in meat rabbits at different growth stages and relationship with average daily gain (ADG)

**DOI:** 10.1186/s12866-020-01797-5

**Published:** 2020-05-14

**Authors:** Shaoming Fang, Xuan Chen, Jiahua Pan, Qiaohui Chen, Liwen Zhou, Chongchong Wang, Tianfang Xiao, Qian Fu Gan

**Affiliations:** 1 College of Animal Science, Fujian Agriculture and Forestry University, Fuzhou, 350002 China; 2 College of Life Science, Fujian Agriculture and Forestry University, Fuzhou, China

**Keywords:** Ira rabbits, Gut microbiota, 16S rRNA, Dynamic distribution, ADG

## Abstract

**Background:**

The mammalian intestinal tract harbors diverse and dynamic microbial communities that play pivotal roles in host health, metabolism, immunity, and development. Average daily gain (ADG) is an important growth trait in meat rabbit industry. The effects of gut microbiota on ADG in meat rabbits are still unknown.

**Results:**

In this study, we investigated the dynamic distribution of gut microbiota in commercial Ira rabbits from weaning to finishing and uncover the relationship between the microbiota and average daily gain (ADG) via 16S rRNA gene sequencing. The results indicated that the richness and diversity of gut microbiota significantly increased with age. Gut microbial structure was less variable among finishing rabbits than among weaning rabbits. The relative abundances of the dominant phyla Firmicutes, Bacteroidetes, Verrucomicrobia and Cyanobacteria, and the 15 predominant genera significantly varied with age. Metagenomic prediction analysis showed that both KOs and KEGG pathways related to the metabolism of monosaccharides and vitamins were enriched in the weaning rabbits, while those related to the metabolism of amino acids and polysaccharides were more abundant in the finishing rabbits. We identified 34 OTUs, 125 KOs, and 25 KEGG pathways that were significantly associated with ADG. OTUs annotation suggested that butyrate producing bacteria belong to the family *Ruminococcaceae* and *Bacteroidales_S24-7_group* were positively associated with ADG. Conversely, *Eubacterium_coprostanoligenes_group*, *Christensenellaceae_R-7_group*, and opportunistic pathogens were negatively associated with ADG. Both KOs and KEGG pathways correlated with the metabolism of vitamins, basic amino acids, and short chain fatty acids (SCFAs) showed positive correlations with ADG, while those correlated with aromatic amino acids metabolism and immune response exhibited negative correlations with ADG. In addition, our results suggested that 10.42% of the variation in weaning weight could be explained by the gut microbiome.

**Conclusions:**

Our findings give a glimpse into the dynamic shifts in gut microbiota of meat rabbits and provide a theoretical basis for gut microbiota modulation to improve ADG in the meat rabbit industry.

## Background

The mammalian intestinal tract harbors large and diverse microbial communities. Due to mutualistic relationships, gut microbiota play crucial roles in host health, metabolism, immunity, and development [1, 2]. Moreover, gut microbiota are dynamic and vary according to many factors, including diet, age, host phylogeny, and gut morphology [3].

Previous studies have demonstrated that genetics, nutrition, and diseases could affect the production performance of food producing animals [4, 5]. Recently, the role of gut microbiota in production performances has received more attention. For instance, Díaz-Sánchez et al. suggested using gut microbiota as biomarkers for prediction of production performance in the selective breeding process of chicken [6]. Warne et al. indicated that manipulation of gut microbiota during critical developmental windows could affect production performance in animals [7]. Hence, it is important to understand the dynamic distribution of gut microbiota in food producing animals at different growth stages and to recognize how microbial taxa and functions affect production performance.

Average daily gain (ADG) is an important index of production performance in commercial meat rabbit breeds as it is related to economic benefits for the meat rabbit industry. It has been reported that gut microbiota is intimately correlated with ADG in meat producing animals. He et al. revealed that the phylum Firmicutes showed a positive association with the ADG of meat duck, while the phylum Proteobacteria exhibited the inverse correlation [8]. Ma et al. indicated that broiler chickens with higher ADG harbored more abundant *Christensenellaceae* and *Caulobacteraceae* in the gut [9]. Jiao et al. found that *Desulfopila* was positively associated with ADG in beef cattle [10]. Ramayo-Caldas et al. suggested that increasing the proportion of *Prevotella* in the gut microbial community could improve porcine ADG [11]. However, the relationship between gut microbiota and ADG in meat rabbits remains unclear.

In this study, we investigated the dynamic distribution of gut microbiota in commercial Ira rabbits from weaning to finishing via high-throughput 16S rRNA gene sequencing. Additionally, we identified microbial taxa and potential functional capacities associated with ADG. Our results not only highlight the shifts and differences of gut microbial communities in meat rabbits at different growth stages, but also provide important information for improving ADG by modulating gut microbiota.

## Results

### Sequencing data and microbial diversity analysis

The 16S rRNA gene sequencing process generated a total of 30,080,332 paired-end reads in both weaning and finishing samples. After sequences filtering steps and chimera removal, a total of 29,742,985 high-quality reads were obtained (15,380,901 clean reads of weaning samples, 14,362,084 clean reads of finishing samples). Based on 97% sequences identity, 1460 and 1586 OTUs were obtained from samples at weaning and finishing, respectively. A total of 2072 OTUs were identified from all samples, with 974 of those defined as core OTUs due to their existence in both weaning and finishing samples (Additional file 1: Fig. S1a). Additionally, 486 OTUs were uniquely identified in weaning samples and 612 unique OTUs were found in finishing samples.

**Fig. 1:**
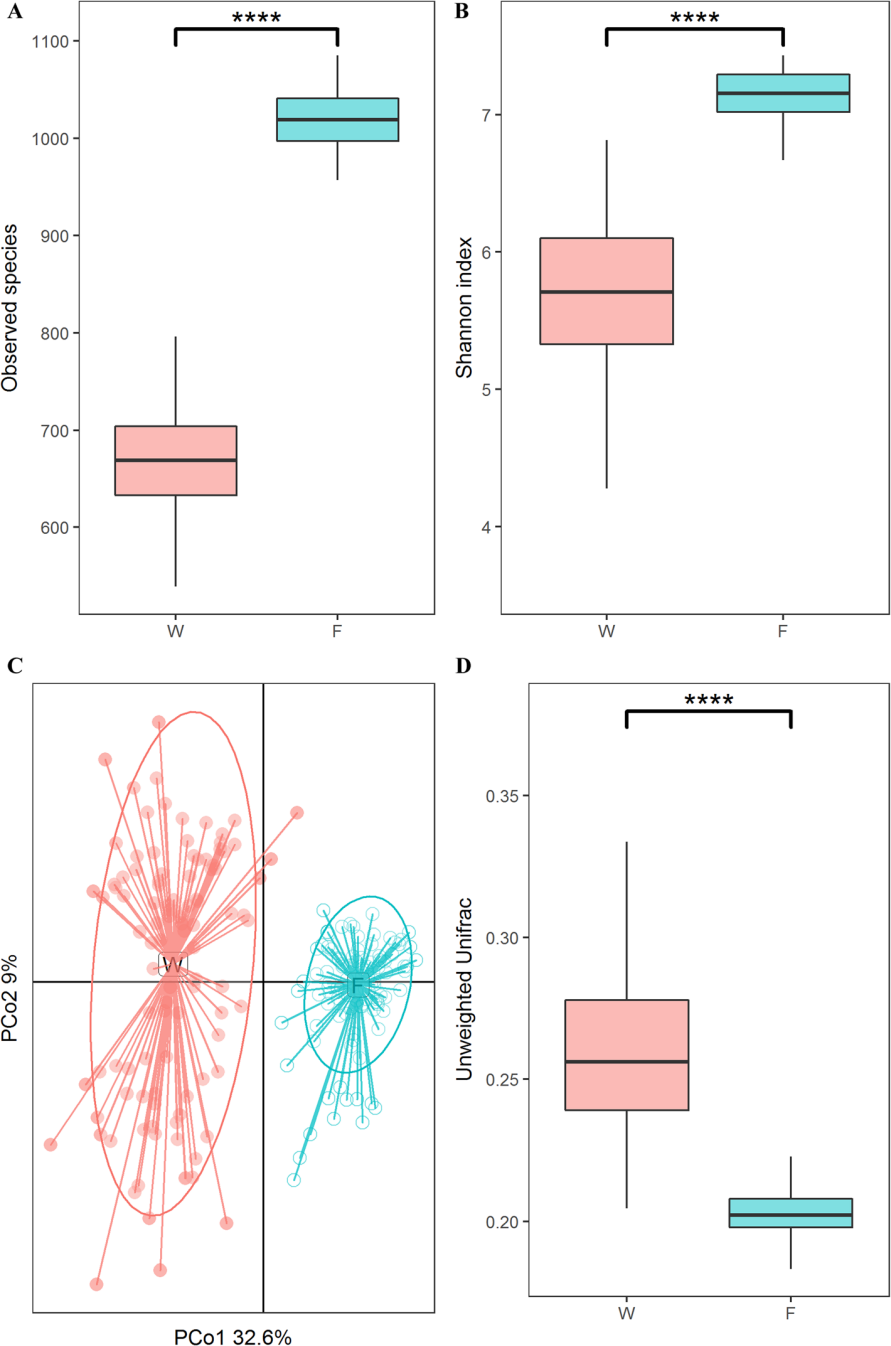
Differences in diversities and structures of gut microbiota between weaning and finishing samples. **a** Observed species index (“W” and “F” represents for weaning and finishing samples, respectively; ***** FDR adjusted *p* < 0.0001). **b** Shannon index. **c** Principal Coordinate Analysis (PCoA) of gut microbial community structures based on Unweighted Unifrac distance. **d** Unweighted Unifrac distance metric

The observed species, Shannon, and Good’s coverage index of alpha diversity were used to analyze the richness and diversity of the gut microbial community. As shown in Fig. 1a, the observed species index of the finishing samples was significantly higher than weaning samples (FDR adjusted *p* < 0.0001). Finishing samples also presented significantly higher Shannon index values than weaning samples did (Fig. 1b, FDR adjusted *p* < 0.0001). In addition, Good’s coverage index values were 0.999 and 0.998 for weaning and finishing rabbits, respectively, all showing excellent coverage (Additional file 1: Fig. S1b).

PCoA was performed to uncover the changes in gut microbial community structures in weaning and finishing samples. Both the unweighted and weighted UniFrac distances indicated that the samples were clustered by age (Fig. 1c, Additional file 1: Fig. S1c). Besides, the unweighted and weighted UniFrac distance metric comparison analysis further revealed less variation in the gut microbiota among finishing samples than among weaning samples (Fig. 1d, Additional file 1: Fig. S1d, FDR adjusted *p* < 0.0001).

### Gut microbiota composition at different taxonomical levels

**Fig. 2:**
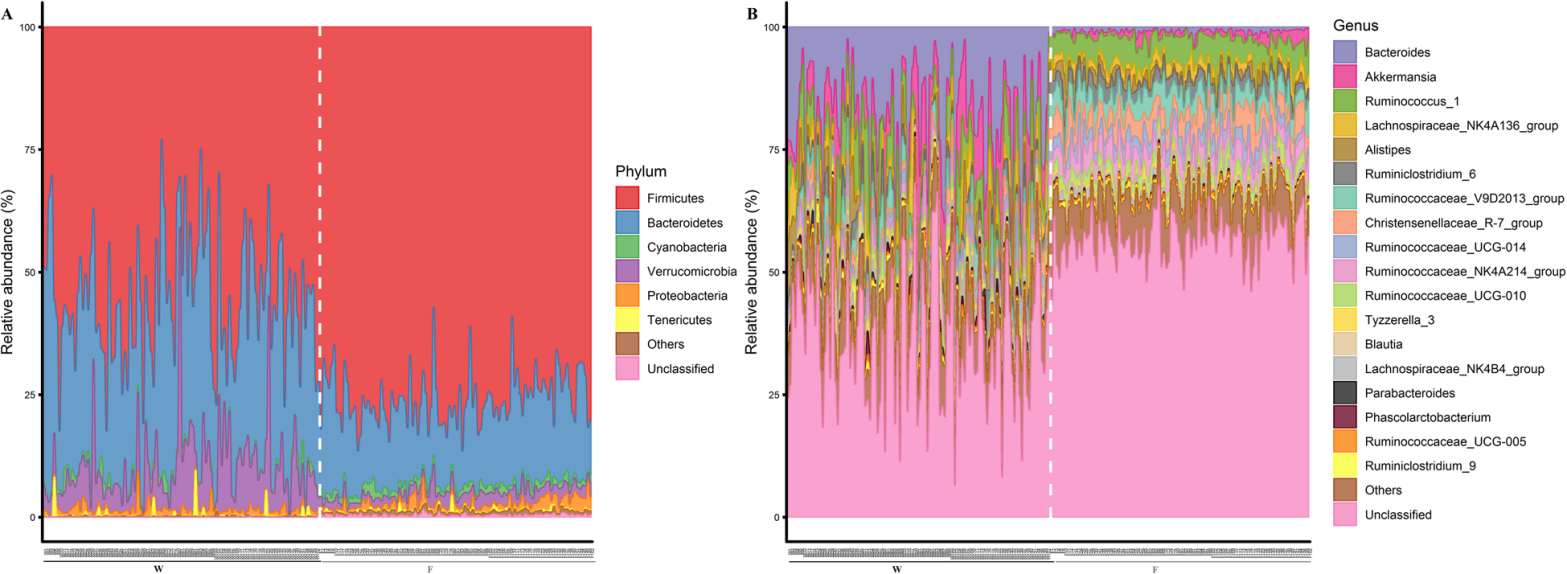
The dynamic distributions of gut microbiota at different taxonomic levels. **a** At phylum level. **b** At genus level. The IDs on the X-axis with the same number but different letters (“W” and “F”) in the two groups represent the same rabbit at weaning and finishing stage, respectively

Gut microbial community composition was analyzed at the phylum and genus level. A total of 10 phyla were shared by weaning and finishing samples, including Actinobacteria, Bacteroidetes, Cyanobacteria, Euryarchaeota, Firmicutes, Lentisphaerae, Proteobacteria, Saccharibacteria, Tenericutes, and Verrucomicrobia (Additional file 1: Fig. S2a). In addition, Synergistetes and Planctomycetes were uniquely found in weaning and finishing samples, respectively. Firmicutes, Bacteroidetes, Verrucomicrobia, Proteobacteria, Cyanobacteria, and Tenericutes were the top six phyla in all samples and they comprised more than 99% of the total sequences (Fig. 2a). Among these, four phyla showed significant differences in relative abundances between weaning and finishing samples (Fig. 2a, Additional file 2: Table S2). The relative abundance of Firmicutes significantly increased from weaning (55.38%) to finishing (75.85%). Conversely, a significant decrease in the relative abundance of Bacteroidetes was observed from weaning (33.39%) to finishing (17.65%). Moreover, weaning samples exhibited a significantly higher abundance of Verrucomicrobia than finishing samples did (8.62% vs. 2.22%, respectively), while finishing samples exhibited a significantly higher abundance of Cyanobacteria than weaning samples did (1.41% vs. 0.82%, respectively).

At the genus level, a total of 87 genera were identified in all samples, including 54 common genera, 24 genera unique to weaning samples, and 9 genera specific to finishing samples (Additional file 1: Figure. S2b). The 18 most abundant genera found in both weaning and finishing samples were *Akkermansia*, *Alistipes*, *Bacteroides*, *Blautia*, *Christensenellaceae_R-7_group*, *Lachnospiraceae_ NK4A136_group*, *Lachnospiracea_NK4B4_group*, *Parabacteroides*, *Phascolarctobacterium*, *Ruminiclostridium_6*, *Ruminiclostridium_9*, *Ruminococcaceae_NK4A214_group*, *Ruminococcaceae_UCG-005*, *Ruminococcaceae _UCG-010*, *Ruminococcaceae_UCG-014*, *Ruminococcaceae_V9D2013_group*, *Ruminococcus_1*, and *Tyzzerella_3* (Fig. 2b). *Akkermansia* belongs to the phylum Verrucomicrobia, *Alistipes*, *Bacteroides*, and *Parabacteroides* are members of the phylum Bacteroidetes, and the other 14 genera derive from phylum Firmicutes. Among these genera, the relative abundances of 15 were significantly changed from weaning to finishing (Additional file 2: Table S2). Similar to the dynamic changes in the relative abundances of the dominant phyla, we found that the relative abundances of *Akkermansia*, *Alistipes*, *Bacteroides*, and *Parabacteroides* significantly declined from weaning to finishing, while the relative abundances of most genera (e.g., *Ruminococcaceae_UCG-010*, *Ruminococcaceae_NK4A214_group*, *Christensenellaceae_R-7_group*, and *Ruminococcaceae _V9D2013_group*) of the phylum Firmicutes significantly increased from weaning to finishing.

### Potential functional profile of gut microbial community

**Fig. 3:**
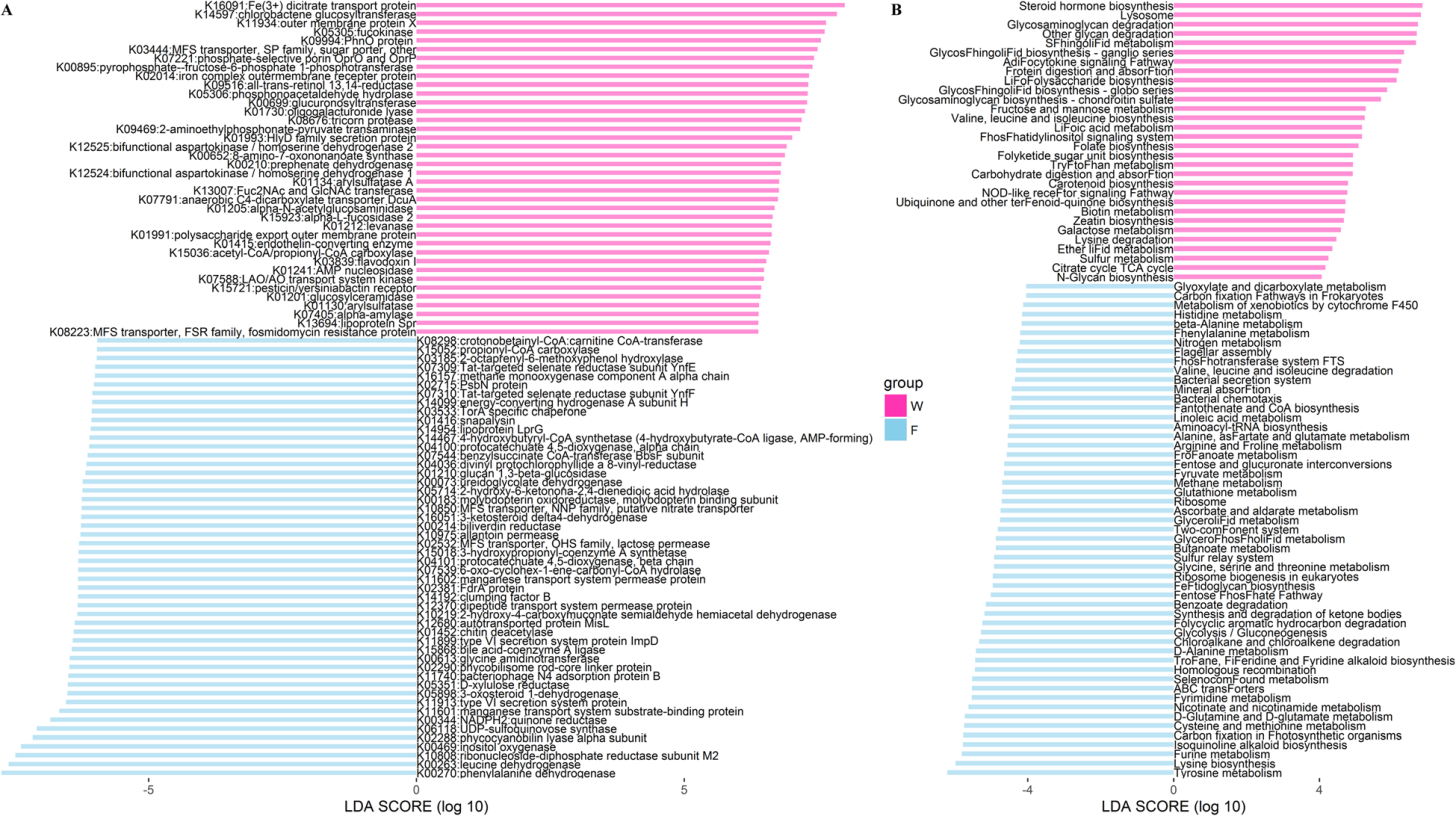
Potential functional capacities of gut microbiota showing different enrichment between weaning and finishing samples. **a** KEGG Orthologs (KOs). **b** KEGG pathways

To investigate the changes in gut microbial functional profiles of weaning and finishing samples, KOs and KEGG pathways were predicted based on 16S rRNA gene sequencing data using Tax4Fun. We identified 6367 common KOs in all samples, while 41 KOs were specific to weaning samples and 137 KOs were unique to finishing samples (Additional file 1: Fig. S3a). In total, 177 KEGG pathways were identified and shared by both weaning and finishing samples (Additional file 1: Fig. S3b). To identify different enrichment of functional capacities between weaning and finishing samples, we performed a LEfSe analysis using the relative abundances of common KOs and pathways. As shown in Fig. 3a (and Additional file 2: Table S3), 88 KOs showed distinct relative abundances between weaning and finishing samples. Thirty-eight KOs enriched in the weaning samples were mostly related to membrane transporters (e.g., K16091, K11934, K03444, K07221, K02014, and K05306), fructose and mannose metabolism (e.g., K05305, K00895, and K01993) and vitamins metabolism (e.g., K09516, K00699, and K00652). Meanwhile, 50 KOs showing higher abundances in the finishing samples were mostly correlated with the metabolism of amino acids (e.g., K00270, K00263, K00613, K10219, and K05714), lipids (e.g., K06118, K05898, K15868, and K16051), polysaccharides (e.g., K05351, K01452, and K01210), and energy (e.g., K02288, K02290, and K02381). Additionally, we identified 83 KEGG pathways differentially enriched in weaning and finishing samples (Fig. 3b, Additional file 2: Table S3). Similarly, KEGG pathways related to the metabolism of glycan (e.g., glycosaminoglycan and other glycan degradation), monosaccharides (e.g., fructose, mannose, and galactose metabolism), vitamins (e.g., lipoic acid and biotin metaboilsm) were more abundant in weaning samples, while KEGG pathways related to the metabolism of amino acids (e.g., cysteine, methionine, arginine and proline metabolism) and short-chain fatty acids (SCFAs) (e.g., butanoate mand propanoate metabolism) were enriched in finishing samples.

### Microbial taxa and potential functional capacities associated with ADG

**Fig. 4:**
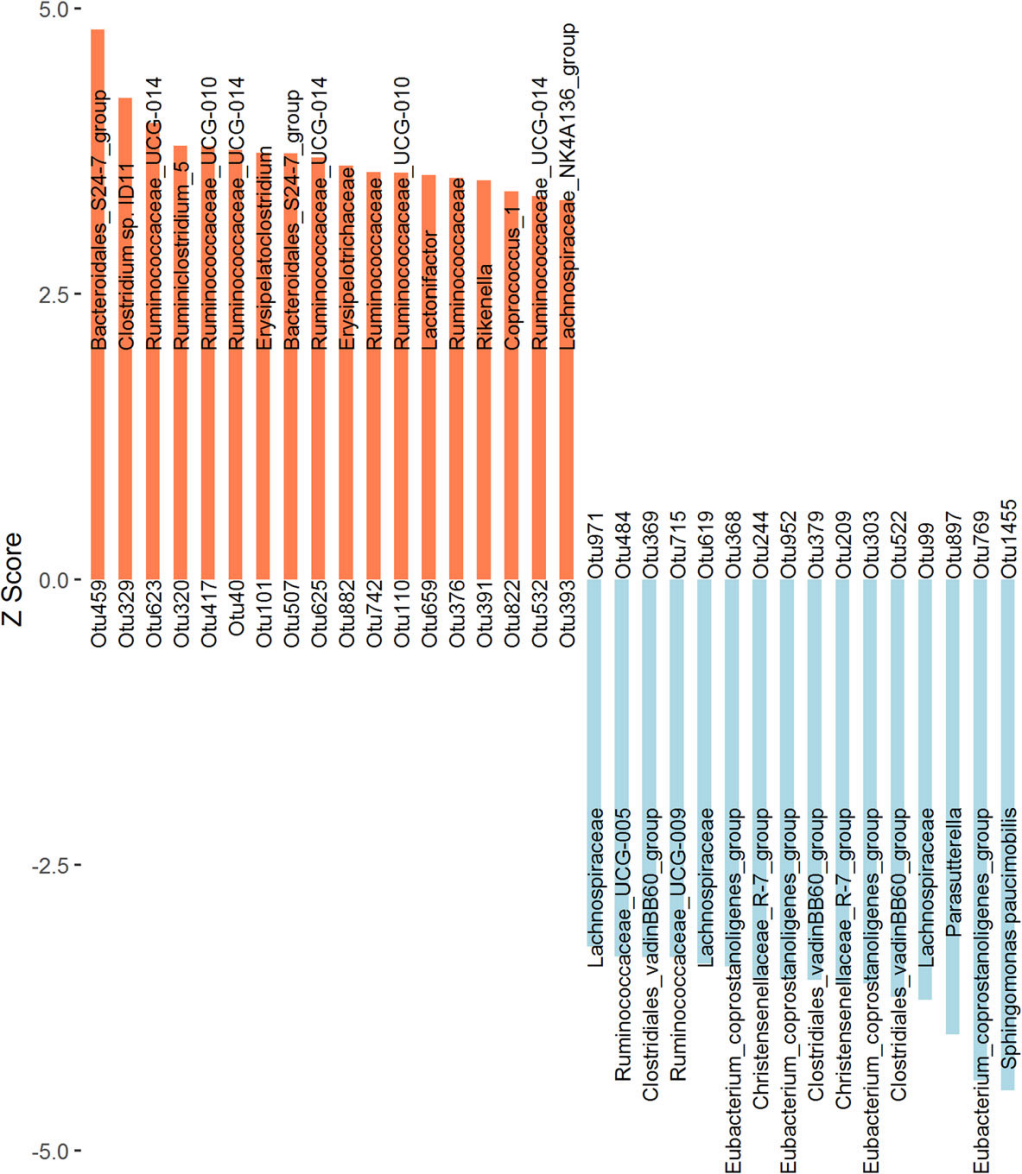
The 34 OTUs showing significant associations with ADG are shown as Z scores. The coral bar represents for positive association, the blue bar corresponds to negative association, and the text on the bar shows the microbial taxa annotated to the OTU

To identify microbial taxa associated with ADG, we performed the two-part model analysis using the ADG phenotypic values adjusted for sex and cage effects, and the relative abundances of OTUs in finishing samples. A total of 34 OTUs were identified that exhibited significant associations with ADG. Among these OTUs, 18 were positively associated with ADG and 16 had negative associations with ADG. We annotated these OTUs to microbial taxa using the SILVA database (Fig. 4, Additional file 2: Table S4), the phylogenetic relationships and abundances of these OTUs were shown in Additional file 1: Fig. S4 and Fig. S5.

Among the positive ADG-associated OTUs, five were annotated to family level, including two OTUs to each of *Bacteroidales_S24-7_group* and *Ruminococcaceae*, and one OTU to *Erysipelotrichaceae*. At the genus level, four OTUs were annotated to *Ruminococcaceae_UCG-014*, two OTUs to *Ruminococcaceae_UCG-010*, and one OTU to each of *Ruminiclostridium_5*, *Erysipelatoclostridium*, *Lactonifactor*, *Rikenella*, *Coprococcus_1*, and *Lachnospiraceae_NK4A136_group.* One OTU was annotated to species Clostridium sp. ID11. Among the negative ADG-associated OTUs, three were annotated to each of the families *Clostridiales_ vadinBB60_group* and *Lachnospiraceae*. Four OTUs were annotated to the genus *Eubacterium_coprostanoligenes_group*, two to the genus *Christensenellaceae_ R-7_group*, and one to each of the genera *Ruminococcaceae_UCG-009*, *Ruminococcaceae_UCG-005*, and *Parasutterella*. At the species level, one OTU was annotated to *Sphingomonas paucimobilis*.

**Fig. 5:**
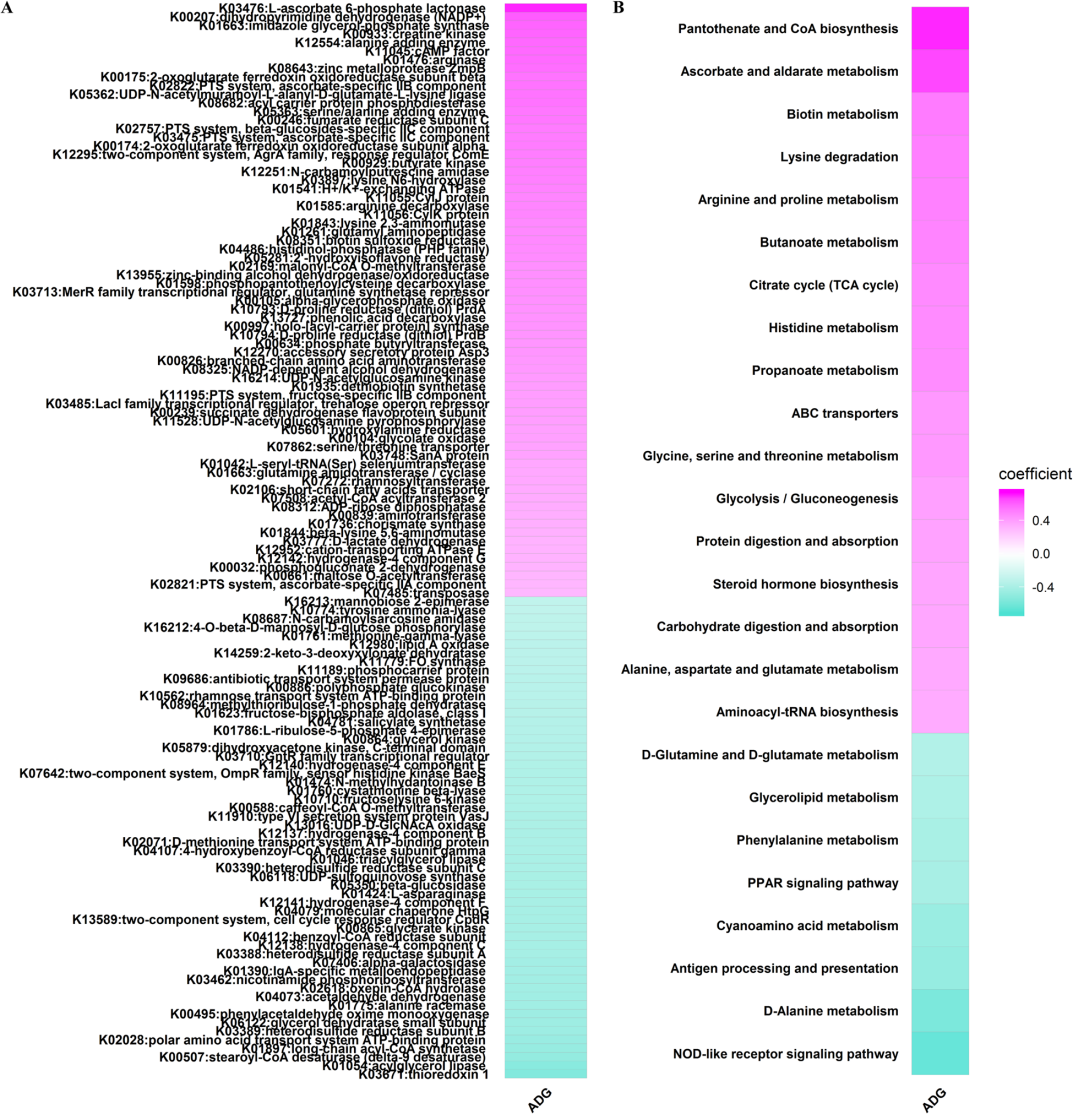
Heatmap of predicted KEGG Orthologs (**a**) and pathways (**b**) significantly associated with ADG (FDR adjusted *p* < 0.05, |r| > 0.4). The correlation coefficient was used to plot

To identify potential functional capacities correlated with ADG, Spearman correlation analysis was performed for the relative abundances of KEGG items and ADG phenotypic values. As shown in Fig. 5a (and Additional file 2: Table S5), 125 KOs were significantly correlated with ADG (FDR adjusted *p* < 0.05, |r| > 0.4). Sixty-nine of 125 KOs showed positive correlations with ADG. Most of these KOs were related to the metabolism of vitamins (e.g., K00207, K02822, K08682, K03475, K08351, K02169, K01598), basic amino acids (e.g., K01663, K00933, K01476, K12251, K03897, K01585, K01843, K04486), and SCFAs (e.g., K00175, K00246, K00174, K00929, K00634, K08325, K00239). Meanwhile, the other 56 KOs that exhibited negative correlations with ADG were mostly associated with lipid metabolism (e.g., K01054, K00507, K01897, K06122, K07406, K00865, K06118), aromatic and non-protein amino acids metabolism (e.g., K00495, K01775, K04073, K02618, K01424, K05350, K00588), and immune responses (e.g., K03671, K03462, K04079). In addition, we identified 25 KEGG pathways showed significant correlations with ADG at |r| > 0.4 and FDR adjusted *p* < 0.05 (Fig. 5b, Additional file 2: Table S5). Among these, pantothenate and CoA biosynthesis, biotin metabolism, lysine degradation, arginine and proline metabolism, butanoate metabolism, propanoate metabolism, and glycine, serine and threonine metabolism were positively correlated with ADG, while NOD-like receptor signaling pathway, D-Alanine metabolism, antigen processing and presentation, cyanoamino acid metabolism, PPAR signaling pathway, phenylalanine metabolism, glycerolipid metabolism, and D-Glutamine and D-glutamate metabolism were negatively associated with ADG.

### Phenotypic variation of ADG explained by gut microbiome

**Fig. 6:**
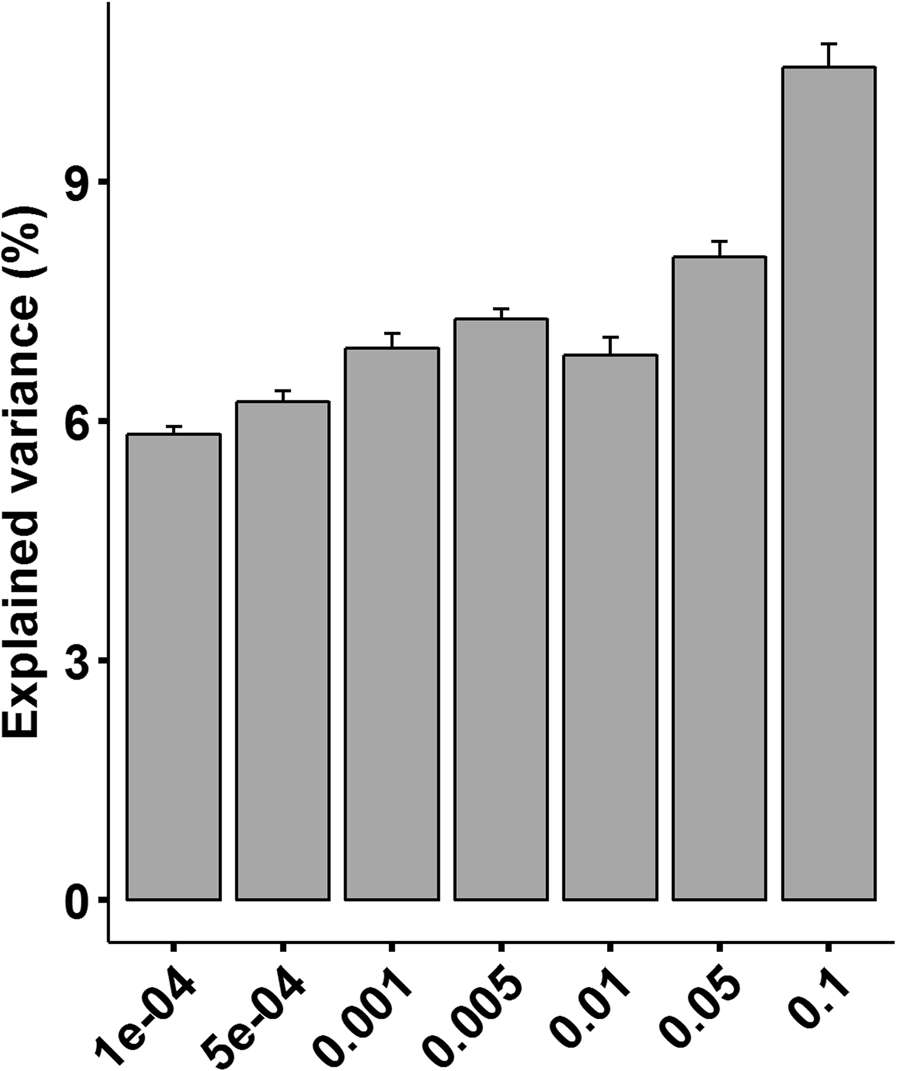
The variation of ADG explained by gut microbiome at different levels of significance

To estimate what proportion of variation in ADG could be explained by the microbiome, we performed 100 times cross-validation analyses at different *p* value thresholds (ranging from 10^− 4^ to 0.1). As shown in Fig. 6, we found that the OTUs identified at *p* = 1.0 × 10^− 4^ could explain 5.83% of the variations in ADG. At *p* = 0.1, the gut microbiome explained 10.42% of the variations in ADG, given that more OTUs were included in the analysis as the threshold increased.

## Discussion

Recently, investigations into mammalian gut microbiota have revealed its vital role in host metabolism, physiology, and immunology. However, few reports have been published on the dynamic distributions of gut microbiota at different growth stages of commercial meat rabbits. ADG is an important growth trait, which is inevitably related to growth stage, but the relation between the gut microbiota and ADG in commercial meat rabbits is still elusive. In the present study, we analyzed the gut microbial diversities and abundances of Ira rabbits at weaning and finishing age. The potential functional profiles of gut microbial communities were then predicted and compared between weaning and finishing rabbits. In addition, we identified microbial taxa and functional capacities associated with ADG.

We found that the richness and diversity of gut microbial communities significantly increased with age (Fig. 1a-b). This result is in agreement with previous findings for animals and humans. Niu et al. reported that gut microbial richness and diversity significantly increased with age in pigs, and suggested that growth stages and conditions were important factors affecting gut microbiota [12]. He et al. conclued that diet and physiological changes may have resulted in the observed increase of the richness and diversity of gut microbiota with increasing age in camels [13]. Additionally, maternal enteric microbes exposure level and breast-milk feeding rate significantly affected gut microbial diversity and richness in the early life was in human infants [14]. Furthermore, we investigated the variation in microbial community structure from weaning to finishing. PCoA analysis suggested that samples were clustered according to age. Unifrac distances further indicated that the gut microbiota of finishing samples showed significantly greater similarity than those of weaning samples (Fig. 1c-d, Additional file 1: Fig. S1c-d). These results could be explained by the fact that all the weaned rabbits fed with the same fatten until finishing, which could minimize the effects of maternal environment and genetics on gut microbiota [15, 16].

As with previous studies [17, 18], we found that Firmicutes, Bacteroidetes, Verrucomicrobia, Proteobacteria, Cyanobacteria, and Tenericutes were the dominant phyla in both weaning and finishing samples. Moreover, the significant increases in the relative abundances of Firmicutes and Cyanobacteria were observed as the rabbits aged, along with a significant decline in the abundances of Bacteroidetes and Verrucomicrobia (Fig. 2a, Additional file 2: Table S2). Zhu et al. also noted that the percentage of Firmicutes increased as rabbits aged and demonstrated that Firmicutes played a vital role in dietary fiber and cellulose degradation [19]. During the fattening period, the rabbits were fed a diet with a high fiber content which should further stimulate the growth of Firmicutes (Additional file 2: Table S1). The intestinal environment became more anaerobic as the host gradually matured, and the increase in the abundances of Cyanobacteria could facilitate obligate anaerobic fermentation and synthesis of vitamins [20]. Bacteroidetes can break down polysaccharides and proteins in breast milk and diet, and facilitate the development of intestinal immune system [21, 22]. Hence, the high prevalence of Bacteroidetes present in the gut microbial community of weaning rabbits fits with feeding pattern (breast milk and solid feed mix-feeding) and immune physiology function development stage. Verrucomicrobia is a relatively newly-defined phylum with largely unknown functions. Interestingly, *Akkermansia muciniphila*, a well-known mucin-degrading bacterium belongs to this phylum, which could improve nutrients extraction during cecotrophy in weaning rabbits. However, its overgrowth in finishing rabbits is closely related to the incidence of epizootic rabbit enteropathy [23, 24]. This could be used to explain the decrease in relative abundances of Verrucomicrobia in healthy finishing rabbits.

As shown in Fig. 2b (and Additional file 2: Table S2), from weaning to finishing, we observed a significant decrease in the relative abundances of genera *Alistipes*, *Bacteroides*, and *Parabacteroides*, which belong to the phylum Bacteroidetes. According to the published reports, *Alistipes* is able to degrade dietary polysaccharides and flourishes in the intestine when host innate immunity at immaturity [25, 26]. *Bacteroides* constitutes essential components of the mammalian intestinal microbiota that is capable of degrading breast milk polysaccharides and stimulating the formation of intestinal mucosa during infancy [27, 28]. *Parabacteroides* is another gut bacteria that participates in breast milk polysaccharides metabolism and its abundance significantly declines with formula milk feeding [29, 30]. Additionally, the abundance of *Akkermansia* (a genus of the phylum Verrucomicrobia) significantly declinesd as rabbits aged, which is in accordance with the results observed for gut microbiota development study in foals [31]*.* By contrast, genera from the phylum Firmicutes, such as *Ruminococcaceae_UCG-010*, *Ruminococcaceae_NK4A214_group*, *Christensenellaceae_R-7_group*, *Ruminococcaceae_V9D2013_group*, and *Ruminococcaceae_UCG-014* exhibited higher abundances in finishing samples. These genera are suggested to exert key roles in dietary cellulose, hemicellulose, and lignocellulose fermentation and SCFAs production [32–34].

Comparison analysis of gut microbial functional capacities indicated that both KOs and KEGG pathways related to the metabolism of monosaccharides and vitamins were enriched in the weaning samples, while those related to the metabolism of amino acids and polysaccharides were more abundant in the finishing samples (Fig. 3, Additional file 2: Table S3). These alternations in the functional profiles should be correlated with the dynamic shifts in gut microbiota at different taxonomic levels. For instance, mannose is an important monosaccharide for protein glycosylation in mammals, and mannose metabolism associated with a higher percentage of *Bacteroides* has been reported in previous studies [35, 36]. Galactose is an essential component of milk oligosaccharides, and a higher relative abundance of *Bacteroides* correlated with the utilization of galactose was observed in the gut microbiome of nursing piglets [37]. Both *Bacteroides* and *Akkermansia* are involved in the metabolism of vitamins, such as biotin and retinol [38, 39]. Previous studies demonstrated that specific bacteria of phylum Firmicutes involved in amino acids (e.g., cysteine and arginine) metabolism could enhance intestinal mucosa immunity and reduce intestinal oxidative stress during the post-weaning period [40, 41]. Besides, both Meale et al. and Ke et al. suggested that dietary polysaccharides metabolism is enhanced with an increase in abundance of Firmicutes [42, 43].

Thirty-four OTUs were significantly associated with ADG (Fig. 4, Additional file 1: Fig. S4 and Fig. S5, Additional file 2: Table S4). Among these, OTUs positively associated with ADG were mostly annotated to members of family *Ruminococcaceae* (e.g., *Ruminococcaceae_UCG-014*, *Ruminiclostridium_5*, and *Ruminococcaceae_UCG-010*) and *Bacteroidales_S24-7_group*. These bacteria are able to produce butyrate by degrading indigestible fibers and polysaccharides [44–46]. Butyrate is not only an energy source for gut microbial growth, but has also been linked to intestinal epithelial cell proliferation and heat shock protein 70 (Hsp70) production [47]. Hsp70 plays an important role in maintaining the functional and structural properties of intestinal epithelial cells in response to pathogens challenge and oxidative stress during weaning transition [48, 49]. These actions of butyrate that promote the intestinal development and health of productive animals are crucial for improving their growth performances [50]. Importantly, butyrate can maintain metabolic homeostasis and modulate immune and inflammatory responses via binding to G protein–coupled receptors FFAR3 and GPR109A, respectively [51, 52], and the optimized metabolic and immune status is beneficial for the growth of farm animals [53]. In addition, butyrate produced by gut microbiota has been found to modulate the growth of animals by inducing secretion of intestinal satiety hormones [e.g., peptide tyrosine tyrosine (PYY) and glucagon-like peptide 1 (GLP1)] and growth hormones [e.g., growth factor insulin like growth factor 1 (IGF-1)] [54, 55]. In contrast, OTUs annotated to *Eubacterium_coprostanoligenes_group*, *Christensenellaceae_R-7_group*, *Parasutterella*, and *Sphingomonas paucimobilis* showed negative associations with ADG. Oral administration of *Eubacterium_coprostanoligenes_group* bacteria in mice reduced body weight by affecting cholesterol metabolism [56, 57]. A Study of the relationship between gut microbiota and ADG in meat ducks indicated that a decline in the abundance of *Eubacterium_coprostanoligenes_group* was accompanied by an increase in ADG [58]. *Christensenellaceae* has long been known to affect host body weight [59]. This may be due to its negative correlation with the ratio of goblet cell to villus height, which affects host’s inherent immunity and nutrient absorption [60, 61]. Additionally, *Parasutterella* and *Sphingomonas paucimobilis* are opportunistic pathogens related to intestinal barrier dysfunction and inflammation, which are detrimental to host growth [62, 63].

We also identified 125 KOs and 25 KEGG pathways showed potential correlations with ADG (Fig. 5, Additional file 2: Table S5). Our results suggested that both KOs and KEGG pathways related to the metabolism of vitamins, basic amino acids, and SCFAs were positively associated with ADG. Vitamins produced by gut microbiota could prevent oxidative stress and anti-inflammation, which maintains intestinal homeostasis and has been linked to promote food intake and body weight gain in mice [64, 65]. In addition, the metabolism of basic amino acids and SCFAs mediated by gut microbiota was shown to affect body weight in pigs. Li et al. found that the metabolism of arginine, butanoate, and propanoate was more active in the gut microbiota of pigs with higher body weight gain [66]. Cheng et al. suggested that gut microbiota modulate increases in the concentrations of acetate, propionate, and butyrate, which contribute to a higher ADG in pigs [67]. In contrast, gut microbiota that participated in aromatic amino acids metabolism and immune response exhibited negative associations with ADG. Previous studies revealed that gut microbiota involved in aromatic amino acids metabolism related to colitis, and producing specific metabolites could reduce host body weight gain [68–70]. Immune response pathways mediated by gut microbiota included the NOD-like receptor signaling pathway and PPAR signaling pathway. These pathways are related to pro-inflammatory cytokines production and immune cells proliferation which exert negative effects on host health and growth [71, 72].

Additionally, we found that the gut microbiome could explain 5.83–10.42% (Fig. 6) of the variation in ADG that effects similar to host genetics on ADG (5.2–9.6%) [73]. This result implies that the effects of gut microbiota should not be underestimated in attempts to improve growth performances of meat rabbits.

## Conclusions

Our study characterized the gut microbiota profiles of weaning and finishing Ira rabbits. Gut microbial richness and diversity increased with age. Significant differences in gut microbial structure were observed between weaning and finishing rabbits. Dynamic shifts in microbial taxa at the phylum and genus level were uncovered between weaning and finishing rabbits. The metagenomic predicted KOs and KEGG pathways exhibited differential enrichment in weaning and finishing rabbits. Our results emphasized the importance of both butyrate producing bacteria and gut microbiota that modulate the metabolism of vitamins, basic amino acids, and SCFAs in promoting the ADG of meat rabbits. In addition, we found that gut microbiome had a similar effect size on ADG as host genetics. Taken together, our results improve our comprehensive understanding of the dynamic distributions of gut microbial communities in meat rabbits, and offer a direction for gut microbiota modulation to improve ADG in the meat rabbit industry.

## Methods

### Animals and sample collection

ADG is a complex quantitative trait [74], therefore, a total of 105 Ira rabbits (53 males and 52 females) were used in the present study derived from Laidewang Animal Husbandry Co., Ltd., Sanming, China. Six to eight pup rabbits per cage were raised with their dam under natural light and room temperature in the same commercial farm. A commercial pellet diet (details are shown in Additional file 2: Table S1) was provided to lactating dams twice a day and pup rabbits had free access to the feed. Pup rabbits were weaned at 28 ± 2 days, at which point one or two rabbits were randomly selected from 90 cages to measure weaning body weight. Hard fecal samples were collected by stimulating the anus. To reduce the effect of differences in initial body weight, 105 rabbits had similar weaning weight (0.9 ± 0.06 kg) were selected and randomly assigned to separate cages (one rabbit per cage) and fed with a fattening diet (details are shown in Table S1) until finishing (72 ± 2 days). Finishing body weight was measured to calculate ADG and hard fecal samples were collected. All rabbits were healthy and had not received antibiotics, anticoccidial drugs, probiotics or prebiotics during experimental period. All fecal samples were dipped into liquid nitrogen for transportation and stored at − 80 °C in the laboratory. At the end of experiments, all rabbits were transported to the local slaughterhouse, stunned with electronarcosis (80 V for 10 s) and quickly bled by cutting the jugular veins and carotid arteries. To avoid the effect of artificial bias, the authors were not involved in the processes of rabbits’ selection, grouping, body weight measurement, and further 16S rRNA gene sequencing.

### DNA extraction and 16S rRNA gene sequencing

Microbial genomic DNA was extracted from feces using the QIAamp Fast DNA Stool Mini Kit (QIAGEN, Hilden, Germany) following the manufacturer’s instructions. Before PCR amplification and sequencing, we assessed the purity and integrity of total DNA by using the Nanodrop ND-2000 spectrophotometer (Thermo Fisher Scientific, Waltham, MA, USA) and 1.5% agarose gel electrophoresis, respectively.

The V3-V4 hypervariable region of the 16S rRNA gene was amplified by the barcoded fusion primers 341F (5′-CCTACGGGNGGCWGCAG-3′) and 806R (5′- GGACTACHVGGGTATCTAAT-3′). The PCR conditions were as follows: initial denaturation step at 95 °C for 3 min, followed by 28 cycles of 95 °C for 30 s, 55 °C for 30 s and 72 °C for 30 s, and a final extension step at 72 °C for 10 min. The PCR products purification was performed using the Agencourt AMPure XP system (Beckman Coulter, Brea, CA, USA). The final DNA libraries were sequenced on a Hiseq-2500 platform (Illumina, San Diego, CA, USA) according to the manufacturer’s instructions.

### 16S rRNA gene sequencing data analysis

Quality control of raw data was performed using QIIME (v.1.9.1), including the removal of the primers, barcodes, and low quality sequences [75]. FLASH (v.1.2.11) was used to merge high-quality paired-end reads into tags [76]. To normalize the sequencing depth, we rarefied the library size of microbial sequences to 40,000 tags per sample before further analysis [77]. Tags were clustered into operational taxonomic units (OTUs) at 97% sequence identity using USEARCH (v.10.0) [78]. We filtered out those OTUs with relative abundance < 0.1% and those that were presented in less than 3% of the experimental rabbits from further analysis. The SILVA database (v.132) was used to assign taxonomic category to OTUs [79]. The alpha and beta diversity indices were calculated using Mothur (v.1.41.1) and QIIME (v.1.9.1), respectively [75, 80]. Potential functional capacities of gut microbiota were predicted using Tax4Fun [81].

### Statistical analysis

Wilcoxon test with false discovery rate (FDR) correction was used to determine differences in observed species, Shannon, Good’s coverage, weighted and unweighted UniFrac distance metric between weaning and finishing rabbits. Principal coordinate analysis (PCoA) was performed using both unweighted and weighted UniFrac distances. The dynamic changes in the relative abundances of microbiota at the phylum and genus level were presented as alluvial diagrams. The numbers of shared OTUs, phyla, genera, KOs, and KEGG pathways were showed as the Venn diagrams. Linear discriminant analysis Effect Size (LEfSe) was used to analyze the differential enrichment of KOs and KEGG pathways in weaning and finishing samples.

After sex and cage effects correction, the residuals of ADG were used for further association analysis between ADG phenotypic values and the relative abundances of OTUs. To overcome the problem of non-normal distribution of OTUs, a two-part model analysis was performed to identify microbial taxa associated with ADG as described previously [82]. Briefly, the two-part model association analysis consisted of binary, quantitative, and meta models. The binary model describes a binomial analysis that tests for associations between ADG and detection of a microbe. The quantitative model tests for associations between ADG and the abundance of microbes, but only the samples where the microbe was present were included in the analysis. The meta model was used to combine the effects of both binary and quantitative analysis. The final association *p* value was assigned from the minimum of *p* values from the binary analysis, quantitative analysis, and meta-analysis. Skewness correction was performed by 1000 × permutation tests. FDR < 0.05 was set as the significance threshold. The phylogenetic relationships of the identified microbes were analyzed using neighbor-joining algorithm and heatmap of their abundances were generated using the SEED2 program [83]. Spearman’s correlation analysis with FDR correction was performed to uncover ADG-associated KOs and KEGG pathways.

To investigate the contribution of the gut microbiome to the variation in ADG, a 100 × cross-validation was performed as described by Fu et al. [84]. We randomly divided the dataset into an 80% discovery dataset and a 20% validation dataset. In the discovery dataset, the two-part model association analysis was performed to identify a number of (n) OTUs that were significantly associated with phenotype at a certain p value and assessed the effect sizes of binary and quantitative features (β_1_ and β_2_) of each OTUs. In the validation dataset, the effect of gut microbiome on ADG (r_m_) for each individual was estimated by an additive model: r_m_ = $\sum \limits_{j=1}^n\left({\beta}_1+{b}_j+{\beta}_2{q}_j\right)$, where b_j_ and q_j_ represents the binary and quantitative feature of j OTU, respectively. We calculated the squared correlation coefficient (R^2^) between r_m_ and the phenotypic value (corrected for sex and cage), which represents the phenotypic variance explained by the gut microbiome. We repeated the cross-validation for 100 times and calculated the average value of the explained variations to ensure validity and stability of the estimation.

## Supplementary information

**Additional file 1: Figure S1.** Comparison of OTUs and other diversity index between weaning and finishing samples. (a) Venn diagram to describe the common and unique OTUs between the two groups. (b) Good coverage index (c) PCoA analysis based on Weighted Unifrac distance. (d) Weighted Unifrac distance metric. **Figure S2.** The Venn diagram representation of the share phyla (a) and genera (b) between weaning and finishing samples. **Figure S3.** The Venn diagram representation of the share KOs (a) and KEGG pathways (b) between weaning and finishing samples. **Figure S4.** The phylogenetic relationships of ADG-associated microbial taxa. The coral and blue tree labels represent for positive and negative ADG associated OTUs. Bootstrap values are shown on the branches. **Figure S5.** The heatmap of abundances of ADG-associated microbial taxa. The coral and blue strips correspond to positive and negative ADG associated OTUs.

**Additional file 2: Table S1.** Composition of pellet diet at different growth stages. **Table S2.** Differences in the relative abundances of dominant phyla and predominant genera. **Table S3.** Different enriched KOs and KEGG pathways between weaning and finishing samples. **Table S4.** OTUs showing significant associations with ADG and taxonomic assignment using SILVA database. **Table S5.** KOs and KEGG pathways significantly associated with ADG.

## Data Availability

We submitted 16S rRNA gene sequencing data to the SRA database in NCBI with accession numbers: SRR9720317, SRR9720318, SRR9720319, SRR8898361, SRR8898364, and SRR8898363.

## Abbreviations

ADG: Average daily gain
16S rRNA: 16S ribosomal ribonucleic acid
OTU: Operational taxonomic unit
PCoA: Principal co-ordinates analysis
LEfSe: Linear discriminant analysis Effect Size
KEGG: Kyoto Encyclopedia of Genes and Genomes
FDR: False discovery rate
SCFAs: Short-chain fatty acids

## References

[1] Valdes AM, Walter J, Segal E, Spector TD (2018). Role of the gut microbiota in nutrition and health. Bmj.

[2] Zhuang L, Chen H, Zhang S, Zhuang J, Li Q, Feng Z (2019). Intestinal microbiota in early life and its implications on childhood health. Genomics Proteomics Bioinformatics.

[3] Yatsunenko T, Rey FE, Manary MJ, Trehan I, Dominguez-Bello MG, Contreras M, Magris M, Hidalgo G, Baldassano RN, Anokhin AP (2012). Human gut microbiome viewed across age and geography. Nature.

[4] Goldansaz SA, Guo AC, Sajed T, Steele MA, Plastow GS, Wishart DS (2017). Livestock metabolomics and the livestock metabolome: a systematic review. PLoS One.

[5] Tait-Burkard C, Doeschl-Wilson A, McGrew MJ, Archibald AL, Sang HM, Houston RD, Whitelaw CB, Watson M (2018). Livestock 2.0 - genome editing for fitter, healthier, and more productive farmed animals. Genome Biol.

[6] Diaz-Sanchez S, Perrotta AR, Rockafellow I, Alm EJ, Okimoto R, Hawken R, Hanning I (2019). Using fecal microbiota as biomarkers for predictions of performance in the selective breeding process of pedigree broiler breeders. PLoS One.

[7] Warne RW, Kirschman L, Zeglin L (2019). Manipulation of gut microbiota during critical developmental windows affects host physiological performance and disease susceptibility across ontogeny. J Anim Ecol.

[8] He J, He Y, Pan D, Cao J, Sun Y, Zeng X (2019). Associations of gut microbiota with heat stress-induced changes of growth, fat deposition, intestinal morphology, and antioxidant capacity in ducks. Front Microbiol.

[9] Ma Y, Wang W, Zhang H, Wang J, Zhang W, Gao J, Wu S, Qi G (2018). Supplemental Bacillus subtilis DSM 32315 manipulates intestinal structure and microbial composition in broiler chickens. Sci Rep.

[10] Jiao S, Cao H, Dai Y, Wu J, Lv J, Du R, Han B (2017). Effect of high-fat diet and growth stage on the diversity and composition of intestinal microbiota in healthy bovine livestock. J Sci Food Agric.

[11] Ramayo-Caldas Y, Mach N, Lepage P, Levenez F, Denis C, Lemonnier G, Leplat JJ, Billon Y, Berri M, Dore J (2016). Phylogenetic network analysis applied to pig gut microbiota identifies an ecosystem structure linked with growth traits. ISME J.

[12] Niu Q, Li P, Hao S, Zhang Y, Kim SW, Li H, Ma X, Gao S, He L, Wu W (2015). Dynamic distribution of the gut microbiota and the relationship with apparent crude fiber digestibility and growth stages in pigs. Sci Rep.

[13] He J, Hai L, Orgoldol K, Yi L, Ming L, Guo F, Li G, Ji R (2019). High-throughput sequencing reveals the gut microbiome of the Bactrian camel in different ages. Curr Microbiol.

[14] Yee AL, Miller E, Dishaw LJ, Gordon JM, Ji M, Dutra S, Ho TTB, Gilbert JA, Groer M (2019). Longitudinal Microbiome Composition and Stability Correlate with Increased Weight and Length of Very-Low-Birth-Weight Infants. mSystems.

[15] Han GG, Lee JY, Jin GD, Park J, Choi YH, Kang SK, Chae BJ, Kim EB, Choi YJ (2018). Tracing of the fecal microbiota of commercial pigs at five growth stages from birth to shipment. Sci Rep.

[16] Zeng B, Han S, Wang P, Wen B, Jian W, Guo W, Yu Z, Du D, Fu X, Kong F (2015). The bacterial communities associated with fecal types and body weight of rex rabbits. Sci Rep.

[17] Mattioli S, Dal Bosco A, Combes S, Moscati L, Crotti S, Cartoni Mancinelli A, Cotozzolo E, Castellini C (2019). Dehydrated Alfalfa and Fresh Grass Supply in Young Rabbits: Effect on Performance and Caecal Microbiota Biodiversity. Animals.

[18] Li P, Yang S, Zhang X, Huang S, Wang N, Wang M, Long M, He J (2018). Zearalenone changes the diversity and composition of Caecum microbiota in weaned rabbit. Biomed Res Int.

[19] Zhu Y, Wang C, Li F (2015). Impact of dietary fiber/starch ratio in shaping caecal microbiota in rabbits. Can J Microbiol.

[20] Di Rienzi SC, Sharon I, Wrighton KC, Koren O, Hug LA, Thomas BC, Goodrich JK, Bell JT, Spector TD, Banfield JF (2013). The human gut and groundwater harbor non-photosynthetic bacteria belonging to a new candidate phylum sibling to cyanobacteria. eLife.

[21] Avershina E, Lundgard K, Sekelja M, Dotterud C, Storro O, Oien T, Johnsen R, Rudi K (2016). Transition from infant- to adult-like gut microbiota. Environ Microbiol.

[22] Hooper LV (2004). Bacterial contributions to mammalian gut development. Trends Microbiol.

[23] Jin DX, Zou HW, Liu SQ, Wang LZ, Xue B, Weu TG, Cai J, Yan TH, Wang ZS (2018). The underlying microbial mechanism of epizootic rabbit enteropathy triggered by a low fiber diet. Sci Rep.

[24] Davies RR, Davies JA (2003). Rabbit gastrointestinal physiology. Vet Clin North Am Exot Anim Pract.

[25] Bauerl C, Collado MC, Zuniga M, Blas E, Perez Martinez G (2014). Changes in cecal microbiota and mucosal gene expression revealed new aspects of epizootic rabbit enteropathy. PLoS One.

[26] Fransen F, van Beek AA, Borghuis T, Meijer B, Hugenholtz F, van der Gaast-de Jongh C, Savelkoul HF, de Jonge MI, Faas MM, Boekschoten MV (2017). The impact of gut microbiota on gender-specific differences in immunity. Front Immunol.

[27] Walker WA, Iyengar RS (2015). Breast milk, microbiota, and intestinal immune homeostasis. Pediatr Res.

[28] Stappenbeck TS, Hooper LV, Gordon JI (2002). Developmental regulation of intestinal angiogenesis by indigenous microbes via Paneth cells. Proc Natl Acad Sci U S A.

[29] Jost T, Lacroix C, Braegger CP, Rochat F, Chassard C (2014). Vertical mother-neonate transfer of maternal gut bacteria via breastfeeding. Environ Microbiol.

[30] Berding K, Wang M, Monaco MH, Alexander LS, Mudd AT, Chichlowski M, Waworuntu RV, Berg BM, Miller MJ, Dilger RN (2016). Prebiotics and bioactive Milk fractions affect gut development, microbiota, and neurotransmitter expression in piglets. J Pediatr Gastroenterol Nutr.

[31] Costa MC, Stampfli HR, Allen-Vercoe E, Weese JS (2016). Development of the faecal microbiota in foals. Equine Vet J.

[32] Liu C, Wu H, Liu S, Chai S, Meng Q, Zhou Z (2019). Dynamic alterations in yak rumen Bacteria community and Metabolome characteristics in response to feed type. Front Microbiol.

[33] Wang B, Ma MP, Diao QY, Tu Y (2019). Saponin-induced shifts in the rumen microbiome and Metabolome of young cattle. Front Microbiol.

[34] Li L, Qu M, Liu C, Pan K, Xu L, OuYang K, Song X, Li Y, Zhao X (2019). Expression of a recombinant Lentinula edodes cellobiohydrolase by Pichia pastoris and its effects on in vitro ruminal fermentation of agricultural straws. Int J Biol Macromol.

[35] Ma Y, Chen C. Prebiotic Functions of Mannose Oligosaccharides Revealed by Microbiomic and Metabolomic Analyses of Intestinal Digesta (P20–017-19). Curr Dev Nutr. 2019;3(Suppl 1):1779.

[36] Sharma V, Smolin J, Nayak J, Ayala JE, Scott DA, Peterson SN, Freeze HH (2018). Mannose alters gut microbiome, prevents diet-induced obesity, and improves host metabolism. Cell Rep.

[37] Guevarra RB, Hong SH, Cho JH, Kim BR, Shin J, Lee JH, Kang BN, Kim YH, Wattanaphansak S, Isaacson RE (2018). The dynamics of the piglet gut microbiome during the weaning transition in association with health and nutrition. J Anim Sci Biotechnol.

[38] Huda MN, Ahmad SM, Kalanetra KM, Taft DH, Alam MJ, Khanam A, Raqib R, Underwood MA, Mills DA, Stephensen CB (2019). Neonatal vitamin a supplementation and vitamin a status are associated with gut microbiome composition in Bangladeshi infants in early infancy and at 2 years of age. J Nutr.

[39] Sugahara H, Odamaki T, Fukuda S, Kato T, Xiao JZ, Abe F, Kikuchi J, Ohno H (2015). Probiotic Bifidobacterium longum alters gut luminal metabolism through modification of the gut microbial community. Sci Rep.

[40] Xu CC, Yang SF, Zhu LH, Cai X, Sheng YS, Zhu SW, Xu JX (2014). Regulation of N-acetyl cysteine on gut redox status and major microbiota in weaned piglets. J Anim Sci.

[41] Qi H, Li Y, Yun H, Zhang T, Huang Y, Zhou J, Yan H, Wei J, Liu Y, Zhang Z (2019). Lactobacillus maintains healthy gut mucosa by producing L-ornithine. Commun Biol.

[42] Ke S, Fang S, He M, Huang X, Yang H, Yang B, Chen C, Huang L (2019). Age-based dynamic changes of phylogenetic composition and interaction networks of health pig gut microbiome feeding in a uniformed condition. BMC Vet Res.

[43] Meale SJ, Li SC, Azevedo P, Derakhshani H, DeVries TJ, Plaizier JC, Steele MA, Khafipour E (2017). Weaning age influences the severity of gastrointestinal microbiome shifts in dairy calves. Sci Rep.

[44] Zhang C, Wu W, Xin X, Li X, Liu D (2019). Extract of ice plant (Mesembryanthemum crystallinum) ameliorates hyperglycemia and modulates the gut microbiota composition in type 2 diabetic Goto-Kakizaki rats. Food Funct.

[45] Evans CC, LePard KJ, Kwak JW, Stancukas MC, Laskowski S, Dougherty J, Moulton L, Glawe A, Wang Y, Leone V (2014). Exercise prevents weight gain and alters the gut microbiota in a mouse model of high fat diet-induced obesity. PLoS One.

[46] Liu J, Hao W, He Z, Kwek E, Zhao Y, Zhu H, Liang N, Ma KY, Lei L, He WS (2019). Beneficial effects of tea water extracts on the body weight and gut microbiota in C57BL/6J mice fed with a high-fat diet. Food Funct.

[47] Lalles JP (2016). Microbiota-host interplay at the gut epithelial level, health and nutrition. J Anim Sci Biotechnol.

[48] Martinez-Montemayor MM, Hill GM, Raney NE, Rilington VD, Tempelman RJ, Link JE, Wilkinson CP, Ramos AM, Ernst CW (2008). Gene expression profiling in hepatic tissue of newly weaned pigs fed pharmacological zinc and phytase supplemented diets. BMC Genomics.

[49] Liu HY, Dicksved J, Lundh T, Lindberg JE (2014). Expression of heat shock proteins 27 and 72 correlates with specific commensal microbes in different regions of porcine gastrointestinal tract. Am J Physiol Gastrointest Liver Physiol.

[50] Bedford A, Gong J (2018). Implications of butyrate and its derivatives for gut health and animal production. Anim Nutr.

[51] Kimura I, Inoue D, Maeda T, Hara T, Ichimura A, Miyauchi S, Kobayashi M, Hirasawa A, Tsujimoto G (2011). Short-chain fatty acids and ketones directly regulate sympathetic nervous system via G protein-coupled receptor 41 (GPR41). Proc Natl Acad Sci U S A.

[52] Macia L, Tan J, Vieira AT, Leach K, Stanley D, Luong S, Maruya M, Ian McKenzie C, Hijikata A, Wong C (2015). Metabolite-sensing receptors GPR43 and GPR109A facilitate dietary fibre-induced gut homeostasis through regulation of the inflammasome. Nat Commun.

[53] Angelakis E (2017). Weight gain by gut microbiota manipulation in productive animals. Microb Pathog.

[54] Fetissov SO (2017). Role of the gut microbiota in host appetite control: bacterial growth to animal feeding behaviour. Nat Rev Endocrinol.

[55] Yan J, Charles JF (2018). Gut microbiota and IGF-1. Calcif Tissue Int.

[56] Li L, Batt SM, Wannemuehler M, Dispirito A, Beitz DC (1998). Effect of feeding of a cholesterol-reducing bacterium, Eubacterium coprostanoligenes, to germ-free mice. Lab Anim Sci.

[57] Kubeck R, Bonet-Ripoll C, Hoffmann C, Walker A, Muller VM, Schuppel VL, Lagkouvardos I, Scholz B, Engel KH, Daniel H (2016). Dietary fat and gut microbiota interactions determine diet-induced obesity in mice. Mol Metab.

[58] Dai SJ, Zhang KY, Ding XM, Bai SP, Luo YH, Wang JP, Zeng QF (2018). Effect of dietary non-phytate phosphorus levels on the diversity and structure of Cecal microbiota in meat duck from 1 to 21 d of age. Poult Sci.

[59] Goodrich JK, Waters JL, Poole AC, Sutter JL, Koren O, Blekhman R, Beaumont M, Van Treuren W, Knight R, Bell JT (2014). Human genetics shape the gut microbiome. Cell.

[60] McCormack UM, Curiao T, Buzoianu SG, Prieto ML, Ryan T, Varley P, Crispie F, Magowan E, Metzler-Zebeli BU, Berry D (2017). Exploring a Possible Link between the Intestinal Microbiota and Feed Efficiency in Pigs. Appl Environ Microbiol.

[61] Chen X, Song P, Fan P, He T, Jacobs D, Levesque CL, Johnston LJ, Ji L, Ma N, Chen Y (2018). Moderate dietary protein restriction optimized gut microbiota and mucosal barrier in growing pig model. Front Cell Infect Microbiol.

[62] Zhang C, Liu Y, Chen S, Qiao Y, Zheng Y, Xu M, Wang Z, Hou J, Wang J, Fan H (2019). Effects of Intranasal Pseudorabies Virus AH02LA Infection on Microbial Community and Immune Status in the Ileum and Colon of Piglets. Viruses.

[63] Fang D, Shi D, Lv L, Gu S, Wu W, Chen Y, Guo J, Li A, Hu X, Guo F (2017). Bifidobacterium pseudocatenulatum LI09 and Bifidobacterium catenulatum LI10 attenuate D-galactosamine-induced liver injury by modifying the gut microbiota. Sci Rep.

[64] Yoshii K, Hosomi K, Sawane K, Kunisawa J (2019). Metabolism of dietary and microbial vitamin B family in the regulation of host immunity. Front Nutr.

[65] Yuan L, Li X, He S, Gao C, Wang C, Shao Y (2018). Effects of natural flavonoid Isoorientin on growth performance and gut microbiota of mice. J Agric Food Chem.

[66] Li Y, Fu X, Ma X, Geng S, Jiang X, Huang Q, Hu C, Han X (2018). Intestinal microbiome-Metabolome responses to essential oils in piglets. Front Microbiol.

[67] Cheng CS, Wei HK, Wang P, Yu HC, Zhang XM, Jiang SW, Peng J (2019). Early intervention with faecal microbiota transplantation: an effective means to improve growth performance and the intestinal development of suckling piglets. Animal.

[68] Call L, Stoll B, Oosterloo B, Ajami N, Sheikh F, Wittke A, Waworuntu R, Berg B, Petrosino J, Olutoye O (2018). Metabolomic signatures distinguish the impact of formula carbohydrates on disease outcome in a preterm piglet model of NEC. Microbiome.

[69] Konopelski P, Konop M, Gawrys-Kopczynska M, Podsadni P, Szczepanska A, Ufnal M (2019). Indole-3-Propionic Acid, a Tryptophan-Derived Bacterial Metabolite, Reduces Weight Gain in Rats. Nutrients.

[70] Zhang XJ, Yuan ZW, Qu C, Yu XT, Huang T, Chen PV, Su ZR, Dou YX, Wu JZ, Zeng HF (2018). Palmatine ameliorated murine colitis by suppressing tryptophan metabolism and regulating gut microbiota. Pharmacol Res.

[71] Hasan AU, Rahman A, Kobori H (2019). Interactions between Host PPARs and Gut Microbiota in Health and Disease. Int J Mol Sci.

[72] Xiao Y, Yan H, Diao H, Yu B, He J, Yu J, Zheng P, Mao X, Luo Y, Chen D (2017). Early gut microbiota intervention suppresses DSS-induced inflammatory responses by deactivating TLR/NLR Signalling in pigs. Sci Rep.

[73] Piles M, David I, Ramon J, Canario L, Rafel O, Pascual M, Ragab M, Sanchez JP (2017). Interaction of direct and social genetic effects with feeding regime in growing rabbits. Genet Sel Evol.

[74] David I, Sanchez JP, Piles M (2018). Longitudinal analysis of direct and indirect effects on average daily gain in rabbits using a structured antedependence model. Genet Sel Evol.

[75] Caporaso JG, Kuczynski J, Stombaugh J, Bittinger K, Bushman FD, Costello EK, Fierer N, Pena AG, Goodrich JK, Gordon JI (2010). QIIME allows analysis of high-throughput community sequencing data. Nat Methods.

[76] Magoc T, Salzberg SL (2011). FLASH: fast length adjustment of short reads to improve genome assemblies. Bioinformatics.

[77] Hughes JB, Hellmann JJ (2005). The application of rarefaction techniques to molecular inventories of microbial diversity. Methods Enzymol.

[78] Edgar RC (2010). Search and clustering orders of magnitude faster than BLAST. Bioinformatics.

[79] Quast C, Pruesse E, Yilmaz P, Gerken J, Schweer T, Yarza P, Peplies J, Glockner FO (2013). The SILVA ribosomal RNA gene database project: improved data processing and web-based tools. Nucleic Acids Res.

[80] Schloss PD, Westcott SL, Ryabin T, Hall JR, Hartmann M, Hollister EB, Lesniewski RA, Oakley BB, Parks DH, Robinson CJ (2009). Introducing mothur: open-source, platform-independent, community-supported software for describing and comparing microbial communities. Appl Environ Microbiol.

[81] Asshauer KP, Wemheuer B, Daniel R, Meinicke P (2015). Tax4Fun: predicting functional profiles from metagenomic 16S rRNA data. Bioinformatics.

[82] He M, Fang S, Huang X, Zhao Y, Ke S, Yang H, Li Z, Gao J, Chen C, Huang L (2016). Evaluating the contribution of gut microbiota to the variation of porcine fatness with the cecum and fecal samples. Front Microbiol.

[83] Vetrovsky T, Baldrian P, Morais D (2018). SEED 2: a user-friendly platform for amplicon high-throughput sequencing data analyses. Bioinformatics.

[84] Fu J, Bonder MJ, Cenit MC, Tigchelaar EF, Maatman A, Dekens JA, Brandsma E, Marczynska J, Imhann F, Weersma RK (2015). The gut microbiome contributes to a substantial proportion of the variation in blood lipids. Circ Res.

