# Supplementary figures and images for: Dynamic distribution of gut microbiota in meat rabbits at different growth stages and relationship with average daily gain (ADG)
Source: BMC Microbiol. 2020 May 14;20:116. doi: 10.1186/s12866-020-01797-5 (PMC7227296; doi:10.1186/s12866-020-01797-5)

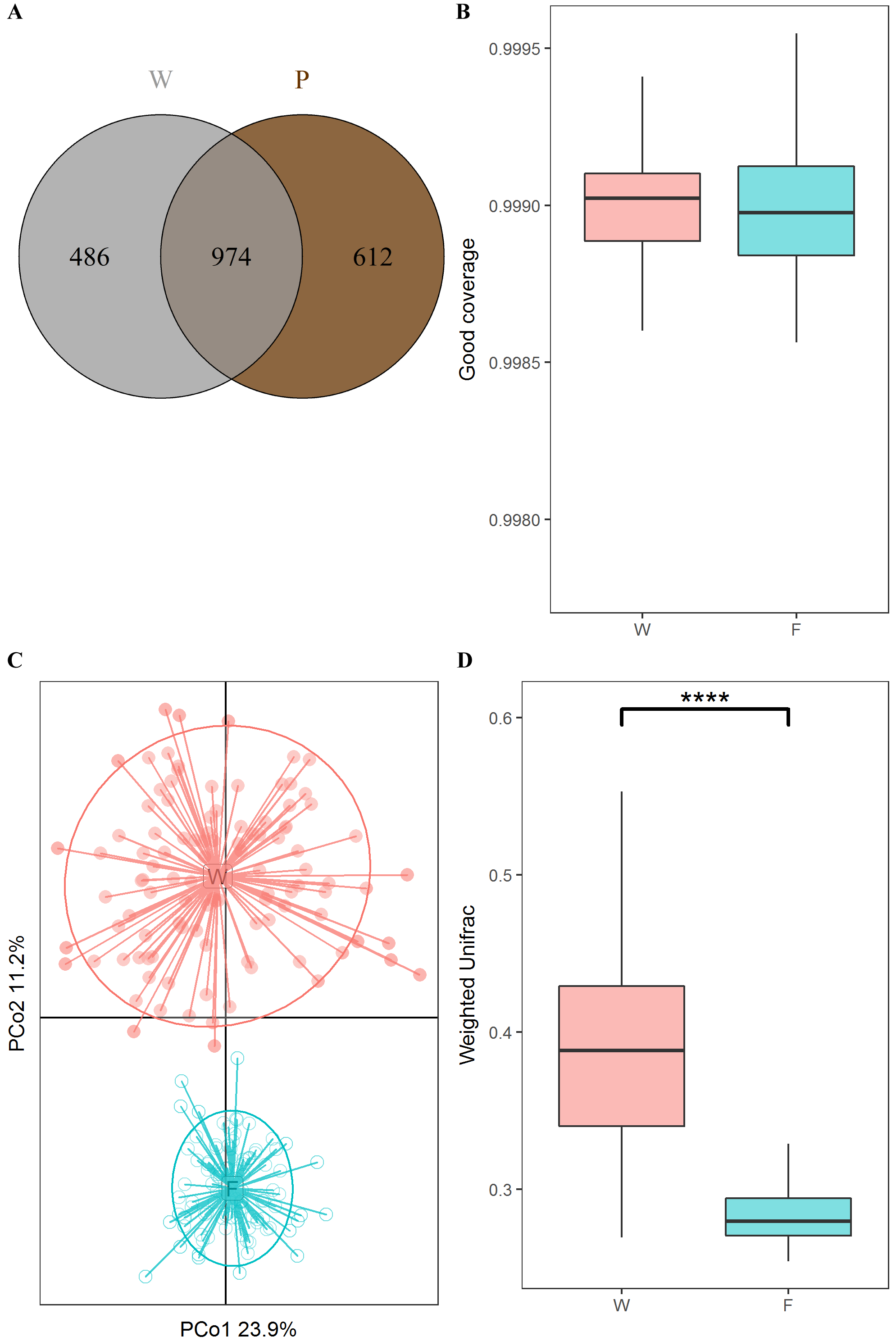

Supplement: Supplementary file 1 — Additional file 1: Figure S1. Comparison of OTUs and other diversity index between weaning and finishing samples. (a) Venn diagram to describe the common and unique OTUs between the two groups. (b) Good coverage index (c) PCoA analysis based on Weighted Unifrac distance. (d) Weighted Unifrac distance metric. Figure S2. The Venn diagram representation of the share phyla (a) and genera (b) between weaning and finishing samples. Figure S3. The Venn diagram representation of the share KOs (a) and KEGG pathways (b) between weaning and finishing samples. Figure S4. The phylogenetic relationships of ADG-associated microbial taxa. The coral and blue tree labels represent for positive and negative ADG associated OTUs. Bootstrap values are shown on the branches. Figure S5. The heatmap of abundances of ADG-associated microbial taxa. The coral and blue strips correspond to positive and negative ADG associated OTUs. [file 12866_2020_1797_MOESM1_ESM.zip › Figure s1.tiff]

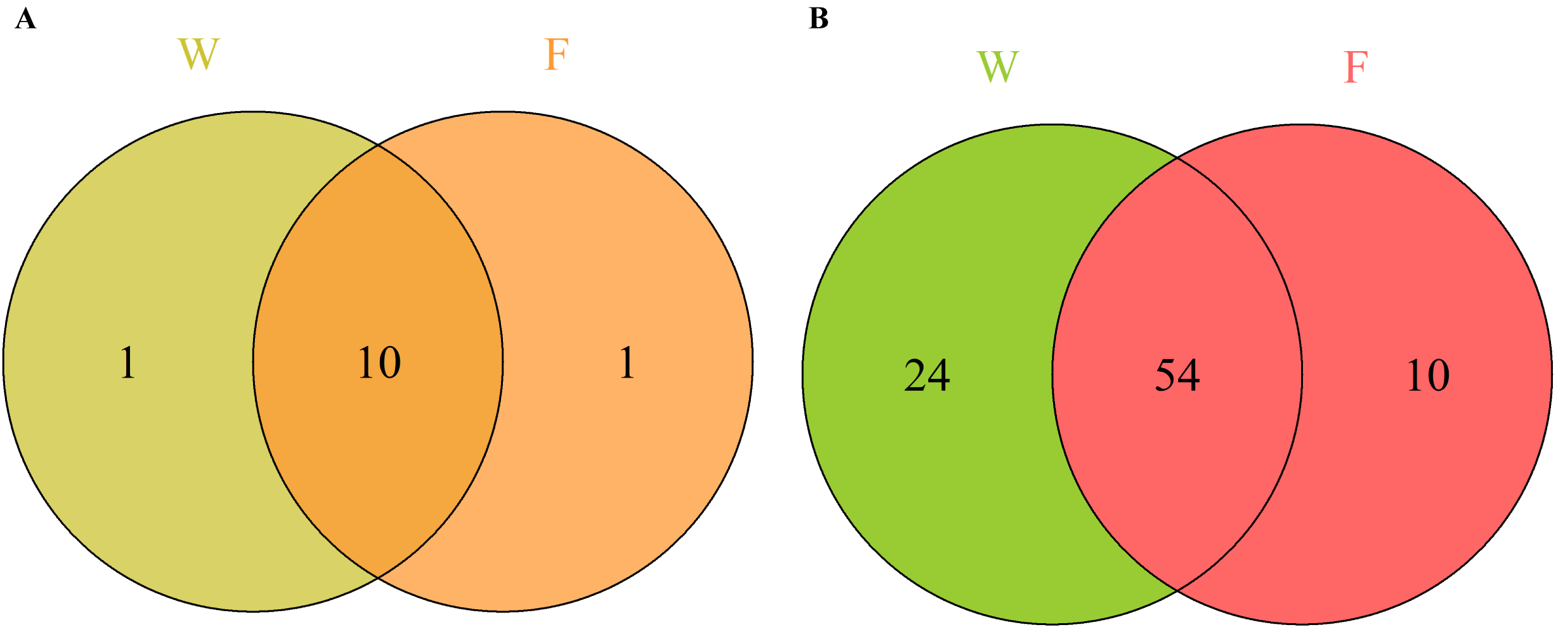

Supplement: Supplementary file 1 — Additional file 1: Figure S1. Comparison of OTUs and other diversity index between weaning and finishing samples. (a) Venn diagram to describe the common and unique OTUs between the two groups. (b) Good coverage index (c) PCoA analysis based on Weighted Unifrac distance. (d) Weighted Unifrac distance metric. Figure S2. The Venn diagram representation of the share phyla (a) and genera (b) between weaning and finishing samples. Figure S3. The Venn diagram representation of the share KOs (a) and KEGG pathways (b) between weaning and finishing samples. Figure S4. The phylogenetic relationships of ADG-associated microbial taxa. The coral and blue tree labels represent for positive and negative ADG associated OTUs. Bootstrap values are shown on the branches. Figure S5. The heatmap of abundances of ADG-associated microbial taxa. The coral and blue strips correspond to positive and negative ADG associated OTUs. [file 12866_2020_1797_MOESM1_ESM.zip › Figure s2.tiff]

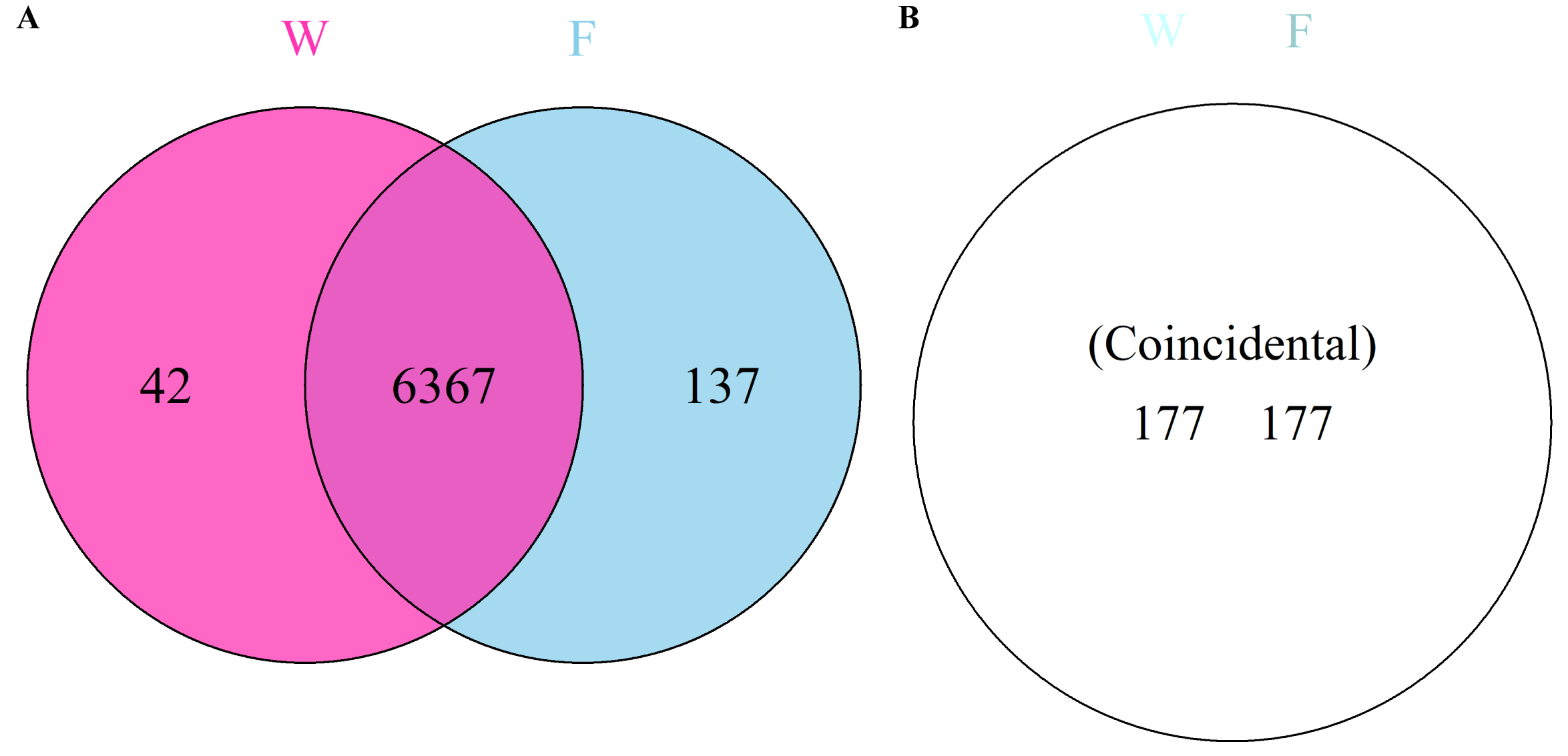

Supplement: Supplementary file 1 — Additional file 1: Figure S1. Comparison of OTUs and other diversity index between weaning and finishing samples. (a) Venn diagram to describe the common and unique OTUs between the two groups. (b) Good coverage index (c) PCoA analysis based on Weighted Unifrac distance. (d) Weighted Unifrac distance metric. Figure S2. The Venn diagram representation of the share phyla (a) and genera (b) between weaning and finishing samples. Figure S3. The Venn diagram representation of the share KOs (a) and KEGG pathways (b) between weaning and finishing samples. Figure S4. The phylogenetic relationships of ADG-associated microbial taxa. The coral and blue tree labels represent for positive and negative ADG associated OTUs. Bootstrap values are shown on the branches. Figure S5. The heatmap of abundances of ADG-associated microbial taxa. The coral and blue strips correspond to positive and negative ADG associated OTUs. [file 12866_2020_1797_MOESM1_ESM.zip › Figure s3.tiff]

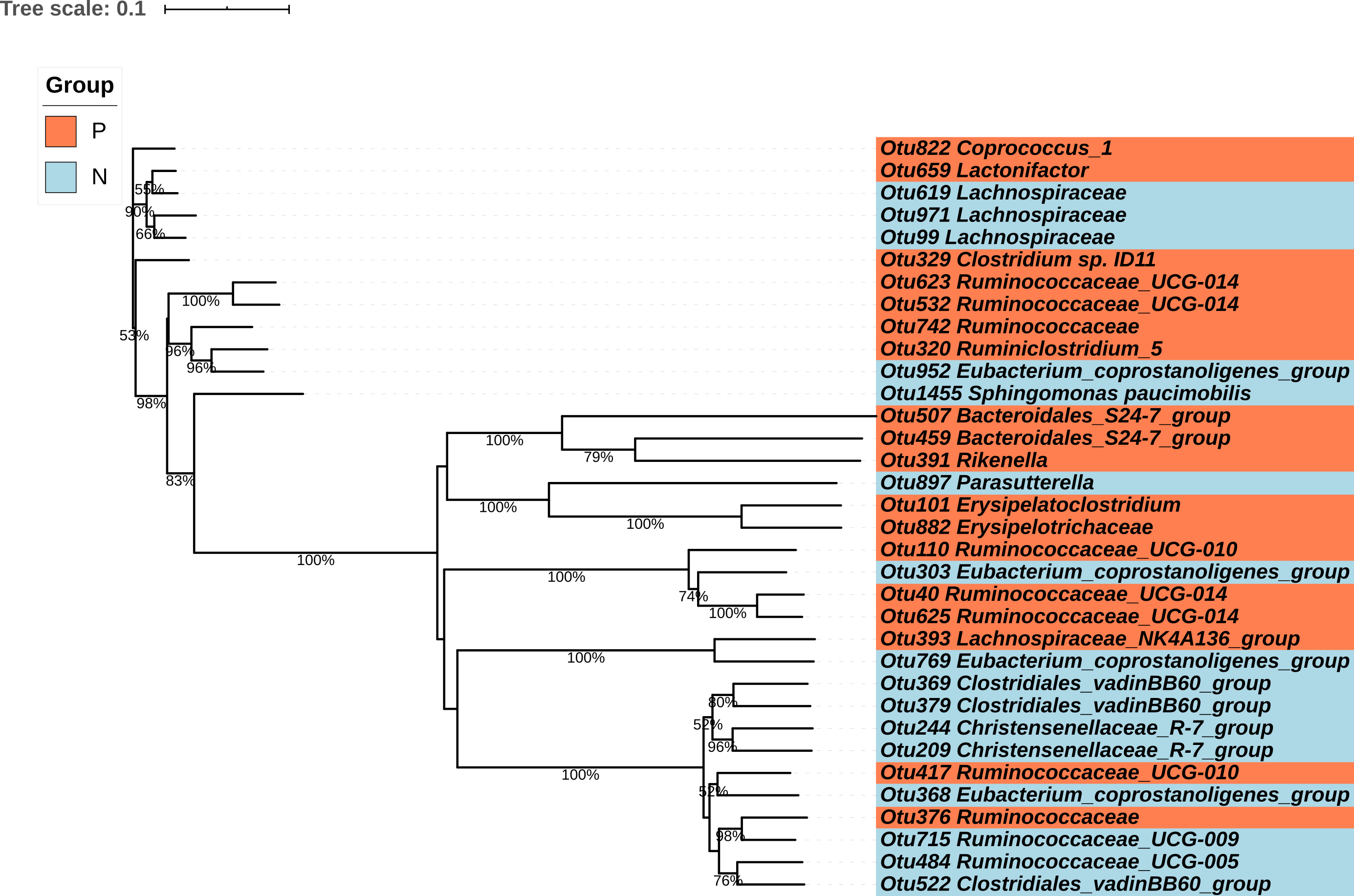

Supplement: Supplementary file 1 — Additional file 1: Figure S1. Comparison of OTUs and other diversity index between weaning and finishing samples. (a) Venn diagram to describe the common and unique OTUs between the two groups. (b) Good coverage index (c) PCoA analysis based on Weighted Unifrac distance. (d) Weighted Unifrac distance metric. Figure S2. The Venn diagram representation of the share phyla (a) and genera (b) between weaning and finishing samples. Figure S3. The Venn diagram representation of the share KOs (a) and KEGG pathways (b) between weaning and finishing samples. Figure S4. The phylogenetic relationships of ADG-associated microbial taxa. The coral and blue tree labels represent for positive and negative ADG associated OTUs. Bootstrap values are shown on the branches. Figure S5. The heatmap of abundances of ADG-associated microbial taxa. The coral and blue strips correspond to positive and negative ADG associated OTUs. [file 12866_2020_1797_MOESM1_ESM.zip › Figure s4.tiff]

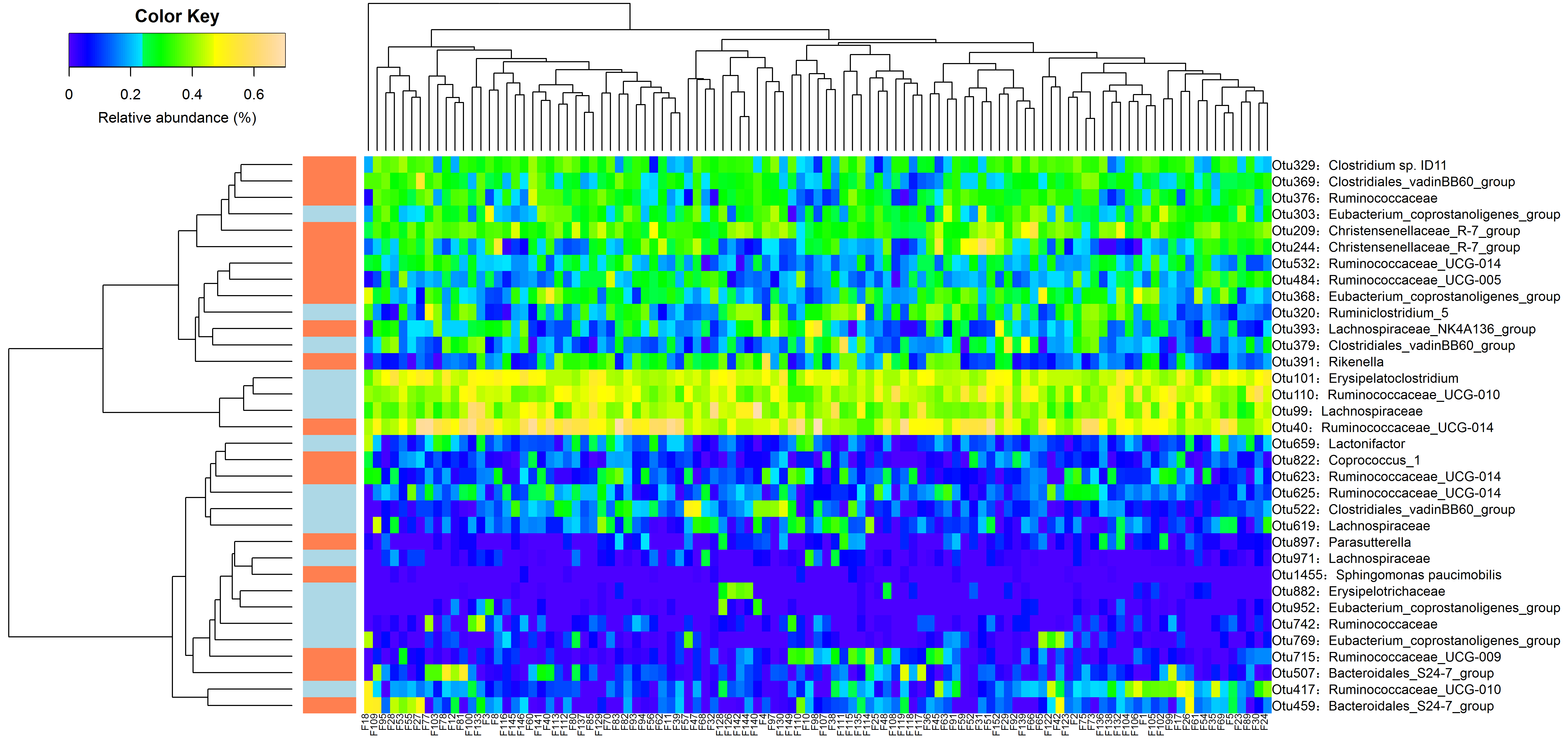

Supplement: Supplementary file 1 — Additional file 1: Figure S1. Comparison of OTUs and other diversity index between weaning and finishing samples. (a) Venn diagram to describe the common and unique OTUs between the two groups. (b) Good coverage index (c) PCoA analysis based on Weighted Unifrac distance. (d) Weighted Unifrac distance metric. Figure S2. The Venn diagram representation of the share phyla (a) and genera (b) between weaning and finishing samples. Figure S3. The Venn diagram representation of the share KOs (a) and KEGG pathways (b) between weaning and finishing samples. Figure S4. The phylogenetic relationships of ADG-associated microbial taxa. The coral and blue tree labels represent for positive and negative ADG associated OTUs. Bootstrap values are shown on the branches. Figure S5. The heatmap of abundances of ADG-associated microbial taxa. The coral and blue strips correspond to positive and negative ADG associated OTUs. [file 12866_2020_1797_MOESM1_ESM.zip › Figure s5.tiff]
